# High dose oral rifampicin to improve survival from adult tuberculous meningitis: A randomised placebo-controlled double-blinded phase III trial (the HARVEST study)

**DOI:** 10.12688/wellcomeopenres.15565.2

**Published:** 2020-08-25

**Authors:** Suzaan Marais, Fiona V Cresswell, Raph L. Hamers, Lindsey H.M. te Brake, Ahmad R. Ganiem, Darma Imran, Ananta Bangdiwala, Emily Martyn, John Kasibante, Enock Kagimu, Abdu Musubire, Kartika Maharani, Riwanti Estiasari, Ardiana Kusumaningrum, Nadytia Kusumadjayanti, Vycke Yunivita, Kogieleum Naidoo, Richard Lessells, Yunus Moosa, Elin M. Svensson, Katherine Huppler Hullsiek, Rob E. Aarnoutse, David R. Boulware, Reinout van Crevel, Rovina Ruslami, David B. Meya

**Affiliations:** 1 Department of Neurology, Inkosi Albert Luthuli Central Hospital, Durban, 4091, South Africa; 2 Infectious Diseases Institute, Mulago College of Health Sciences, Kampala, PO Box 22418, Uganda; 3 Clinical Research Department, London School of Hygiene & Tropical Medicine, London, WC1E 7HT, UK; 4 MRC-UVRI, London School of Hygiene & Tropical Medicine Uganda Research Unit, Entebbe, Uganda; 5 Eijkman-Oxford Clinical Research Unit, Jakarta, Indonesia; 6 Centre for Tropical Medicine and Global Health, Nuffield Department of Medicine, University of Oxford, Oxford, UK; 7 Department of Pharmacy, Radboud Institute for Health Sciences, Radboud University Medical Centre, Nijmegen, The Netherlands; 8 Department of Neurology, Faculty of Medicine, Universitas Padjadjaran/ Hasan Sadikin Hospital, Bandung, 40161, Indonesia; 9 Infectious Disease Research Centre, Faculty of Medicine, Universitas Padjadaran, Bandung, 40161, Indonesia; 10 Department of Neurology, Faculty of Medicine, Universitas Indonesia, Dr Cipto Mangukusumo Hospital, Jakarta, 10430, Indonesia; 11 Division of Biostatistics, School of Public Health, University of Minnesota, Minneapolis, MN, 55455, USA; 12 Department of Microbiology, Faculty of Medicine, Universitas Indonesia, Dr Cipto Mangukusumo Hospital, Jakarta, 10430, Indonesia; 13 Centre for the AIDS programme of research in South Africa (CAPRISA), Doris Duke Medical Research Institute, Durban, 4041, South Africa; 14 CAPRISA-MRC HIV-TB Pathogenesis and Treatment Research Unit, Doris Duke Medical Research Institute, University of KwaZulu Natal, Durban, South Africa; 15 KwaZulu-Natal Research Innovation and Sequencing Platform, Nelson R Mandela School of Medicine, University of KwaZulu-Natal, Durban, 4001, South Africa; 16 Department of Infectious Diseases, Division of Internal Medicine, Nelson R Mandela School of Medicine, University of KwaZulu-Natal, Durban, 4013, South Africa; 17 Department of Pharmaceutical Biosciences, Uppsala University, Uppsala, Sweden; 18 Division of Medicine, University of Minnesota, Minneapolis, MN, 55455, USA; 19 Department of Internal Medicine, Radboud University Nijmegen Medical Centre, Nijmegen, The Netherlands; 20 Department of Biomedical Sciences, Division of Pharmacology and Therapy, Faculty of Medicine, Universitas Padjadjaran, Bandung, 40161, Indonesia

**Keywords:** Tuberculous Meningitis, TB, rifampicin, Xpert Ultra, HIV, treatment, RCT

## Abstract

**Background: **Tuberculous meningitis (TBM), the most severe form of tuberculosis (TB), results in death or neurological disability in >50%, despite World Health Organisation recommended therapy. Current TBM regimen dosages are based on data from pulmonary TB alone. Evidence from recent phase II pharmacokinetic studies suggests that high dose rifampicin (R) administered intravenously or orally enhances central nervous system penetration and may reduce TBM associated mortality. We hypothesize that, among persons with TBM, high dose oral rifampicin (35 mg/kg) for 8 weeks will improve survival compared to standard of care (10 mg/kg), without excess adverse events.

**Protocol: **We will perform a parallel group, randomised, placebo-controlled, double blind, phase III multicentre clinical trial comparing high dose oral rifampicin to standard of care. The trial will be conducted across five clinical sites in Uganda, South Africa and Indonesia. Participants are HIV-positive or negative adults with clinically suspected TBM, who will be randomised (1:1) to one of two arms: 35 mg/kg oral rifampicin daily for 8 weeks (in combination with standard dose isoniazid [H], pyrazinamide [Z] and ethambutol [E]) or standard of care (oral HRZE, containing 10 mg/kg/day rifampicin). The primary end-point is 6-month survival. Secondary end points are: i) 12-month survival ii) functional and neurocognitive outcomes and iii) safety and tolerability. Tertiary outcomes are: i) pharmacokinetic outcomes and ii) cost-effectiveness of the intervention. We will enrol 500 participants over 2.5 years, with follow-up continuing until 12 months post-enrolment.

**Discussion:** Our best TBM treatment still results in unacceptably high mortality and morbidity. Strong evidence supports the increased cerebrospinal fluid penetration of high dose rifampicin, however conclusive evidence regarding survival benefit is lacking. This study will answer the important question of whether high dose oral rifampicin conveys a survival benefit in TBM in HIV-positive and -negative individuals from Africa and Asia.

**Trial registration:** ISRCTN15668391 (17/06/2019)

## Introduction

### Disease burden and prognosis

Tuberculosis (TB) was the leading cause of mortality worldwide from a single infectious agent and, in 2018, 10 million cases were reported globally ^1^. Tuberculous meningitis (TBM) accounts for approximately 1% of global TB cases but has a profound impact due to its high morbidity and mortality ^2^; 19–28% of HIV-negative ^3^ and 40–58% of HIV-positive patients die during treatment ^4, 5^ and half of survivors suffer neurologic disability ^6^. This results in a high burden to health care systems and caregivers with prolonged hospital admissions and rehabilitation periods. There is estimated to be 100 000 cases worldwide annually, but due to inaccurate diagnostics and scarce epidemiological data in many TB endemic regions this figure is likely an underestimate ^2, 7^. TBM disproportionately affects those in high TB prevalence countries, children under 5 years of age and those with HIV coinfection ^2^. It is the second leading cause of meningitis in in hospitalised adults in South Africa and Uganda ^8, 9^, and the leading cause of adult meningitis among those admitted to neurology wards in Indonesia ( Table 1) ^10^.

**Table 1.** Prevalence of meningitis aetiologies in Harvest trial site countries.

Hospital Location	Country	Sample Size	HIV- positive	Meningitis Aetiology Distribution			
				Bacterial	TBM	Cryptococcal	Other / Unknown
Kampala / Mbarara ^8^	Uganda	416	98%	4%	8%	59%	29%
Cape Town ^9^	S. Africa	1,737	96%	19%	13%	30%	38%
Jakarta ^10^	Indonesia	274	54%	0%	34%	5%	61%

TBM = tuberculous meningitis

### Treatment

Current World Health Organisation (WHO) recommended therapy consists of rifampicin, isoniazid, pyrazinamide and ethambutol for two months (intensive phase), followed by rifampicin and isoniazid alone to complete 9–12 months (continuation phase). Factors known to improve survival include early TB treatment initiation and adjunctive corticosteroids ^11^. Nevertheless, morbidity and mortality remains unacceptably high ^12^, likely in part due to delays in seeking medical care, diagnosis and initiation of treatment. Current drug choice, dose and routes of administration for TBM treatment are based on principles used for pulmonary TB (PTB) ^13, 14^. However, unlike PTB, antibiotics to treat TBM must cross the blood-brain barrier (BBB) or blood-cerebrospinal fluid-barrier (BCSFB). Therefore, another possible contributory factor towards mortality in TBM may be poor central nervous system (CNS) penetration of vital TB drugs ^15^.

**Figure 1.**
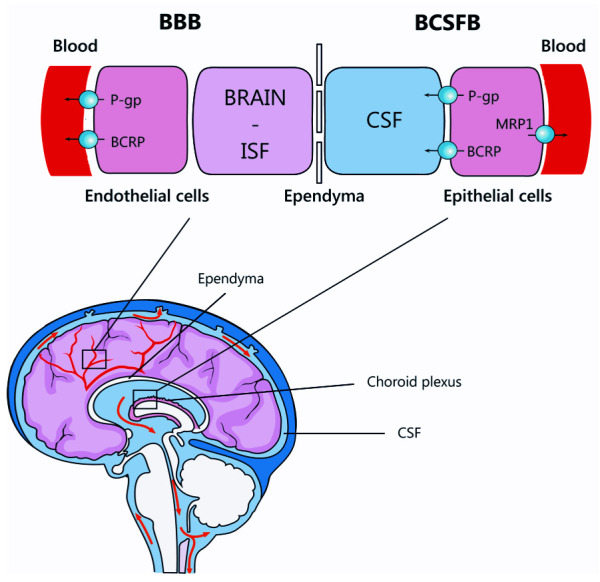
Schematic overview of the blood-brain barrier (brain endothelial cells) and the blood-CSF barrier (choroid plexus epithelial cells). Reproduced with permission from Cresswell *et al.* ^21^.

Factors affecting drug entry into the brain and CSF include lipid solubility, ionisation, molecular weight, protein binding and processes of active transport. Meningeal inflammation also impacts cell tight junction integrity and therefore drug levels in the brain and CSF. Due to variable TB drug penetration across the BCSFB and BBB ( Figure 1), optimal treatment regimens for PTB may not be most effective for TBM. It follows, that altering drug selection, dosing and route of administration may improve treatment outcomes by ensuring adequate delivery to the site of disease and early mycobacterial activity in the CNS ^15^.

### Limitations of standard dose rifampicin

Rifampicin forms the backbone of TBM treatment, and is believed to be the most critical drug in the treatment of TB, as supported by the near-universal fatal outcome reported in patients infected with rifampicin and isoniazid resistant *Mycobacterium tuberculosis* strains (multidrug resistant (MDR)-TB) ^16, 17^. While some excess mortality may be explained by delayed diagnosis of rifampicin resistance, mortality remains greater than 75% in patients diagnosed early enough that second-line drugs can be initiated ^17, 18^.

In plasma, the historical recommended target peak concentrations of rifampicin are 8-24mg/L. This historical target is based on rifampicin concentrations typically observed in healthy adults (i.e. the reference range after a dose of 10mg/kg orally), and is not optimized based on pharmacokinetic-pharmacodynamic (PK-PD) data ^19^. In is now accepted that total exposure to TB drugs, or the area under the 24-h concentration–time curve (AUC _0–24_), is more relevant to the efficacy of first-line TB drugs. Ideally, AUC _0–24_ is considered together with the minimum inhibitory concentration (MIC) of the mycobacteria to yield an AUC _0–24_/MIC ratio that is supposed to best predict response in murine models ^20^. A large meta-analysis of rifampicin pharmacokinetic data amongst TB patients found that the mean plasma rifampicin peak concentration at steady state is only 5.79mg/L (below the historical target concentration) and doses of >25mg/kg are needed to achieve the PK-PD target derived from murine studies (AUC/MIC >271) ^22^. Animal and human pharmacokinetic-pharmacodynamic (PK-PD) studies suggest that plasma rifampicin exposure at current dosing (10 mg/kg) provides exposure that it is sub-optimal in most patients ^23^.

CSF rifampicin concentration is reported to approximately 12% of total (protein-bound plus unbound) plasma concentrations and approximately 50% based on estimated unbound concentrations ^24– 26^. The standard dose of rifampicin is known to often result in CSF concentration below or around the *M. tuberculosis* minimum inhibitory concentration (MIC) for rifampicin of 0.2–0.4 mg/L ^15^. In fact, CSF rifampicin concentrations were below the level of detection in approximately 67% of Indonesian patients with TBM receiving standard oral adult dose (8–10mg/kg) ^27^.

Additional factors may affect rifampicin exposure in TBM patients. For example, HIV co-infection may influence the concentrations of rifampicin achieved in the plasma and CSF. A meta-analysis of rifampicin pharmacokinetic data from 931 individuals spanning 30 years concluded that HIV status affects the total exposure to rifampicin in the early days of treatment (AUC 37.2 mg.h/L in HIV-positive versus 56.7 mg.h/L in HIV-negative after standard-dose (10 mg/kg) rifampicin, p=0.003) ^22^. HIV associated enteric infections and enteropathy causing malabsorption and increased systemic drug clearance secondary to low bodyweight may disproportionately affect drug concentration in these individuals. In addition, these patients are often critically ill on presentation and receive drugs via a nasogastric tube, which may further affect drug delivery.

The extent to which blood and CSF rifampicin concentrations relate to brain tissue exposure is also unclear. Interesting recent data on brain (intralesional) rifampicin pharmacokinetics, derived from a rabbit model of experimentally-induced TBM using serial noninvasive dynamic 11C-rifampin positron emission tomography (PET) over 6 weeks, has shed some light on this matter. Rifampin penetration into infected brain lesions is limited, spatially heterogeneous, and decreases rapidly as early as 2 weeks into treatment. Moreover, rifampin concentrations in the cerebrospinal fluid did not correlate well with those in the brain lesions. First-in-human 11C-rifampin PET performed in a patient with TBM confirmed these findings. PK modeling using this data predicted that higher rifampin doses (≥30 mg/kg) are required to achieve adequate intralesional concentrations in TBM ^28^.

### High dose rifampicin in pulmonary TB

Trials from both the PanACEA consortium and Peru have shown encouraging results supporting both the efficacy and safety of high dose rifampicin up to a dose of 40 mg/kg ^29– 32^. The PanACEA multi-arm multistage trial showed that time to stable culture conversion in liquid media was faster in the 35 mg/kg rifampicin group than in the control group (median 48 days vs 62 days, adjusted hazard ratio 1·78; 95% confidence interval (CI) 1·22–2·58, p=0·003), but not in other experimental arms ^33^. In an additional analysis, increasing rifampicin exposure was also associated with shortened time to stable culture conversion. The effect did not plateau, indicating that doses >35 mg/kg could be yet more effective ^32^.

High-doses rifampicin was also associated with a greater estimated fall in M. tuberculosis bacillary load in sputum of patients with pulmonary TB at 2 weeks follow up, i.e. high-dose rifampicin showed increased ‘early bactericidal activity’ ^29, 30^. Recent analyses of one of these latter trials (the PanACEA HIGHRIF1 trial) included doses up to 40 mg/kg, demonstrating a further effect of rifampicin exposure on early bactericidal activity and clinical trial simulations showed greater early bactericidal activity for 50 mg/kg rifampicin ^34, 35^. Recent evaluations of administration of this 50 mg/kg rifampicin dose show that this dose is not well-tolerated. The maximum tolerable dose for rifampicin in PTB is therefore set at 40 mg/kg daily (R. Aarnoutse and L. te Brake, personal communication, data from the PanACEA HIGHRIF1 trial).

In summary, data from pulmonary TB have shown that higher rifampicin doses, starting with at least 20 mg/kg daily but certainly at 35 mg/kg orally, result in strongly increased systemic exposures to rifampicin, were safe, tolerated, and resulted in increased response, as reflected in improved early bactericidal activity and shorter time to culture conversion. These lessons learnt in pulmonary TB are relevant to TBM treatment, considering the key role of rifampicin in TBM treatment and the limited CSF penetration seen at standard dose.

### Situation of equipoise

Three Indonesian phase II trials have investigated the safety/tolerability of high dose rifampicin to treat TBM. In 2013, Ruslami *et al*., randomised 60 adults with TBM to receive 2 weeks of intensified treatment with either standard or 33% higher intravenous rifampicin dose (13mg/kg), and then either 400mg, 800mg or no moxifloxacin in a factorial design ^24^. They found that a 33% increase in rifampicin dose led to a three-fold increase in geometric mean plasma area under the curve (AUC _0–6h_), maximum plasma concentration (C _max_) and maximum CSF concentration (C _highest_). Although patient numbers were small, there was a significantly lower 6-month mortality (HR 0.42, 95% CI0.2 to 0.91; p=0.03), a quicker resolution of coma (median 4 vs 5 days) and a higher proportion of patients with a complete neurological recovery (31% vs 13%) in the high dose intravenous rifampicin arm. PK-PD analysis revealed that patients who survived in the first two weeks had a significantly higher rifampicin plasma AUC _0–6h_, plasma C _max _and CSF C _highest_, with a strong concentration-effect relationship ^36^. The effect of rifampicin was similar in both moxifloxacin groups, although statistical power was too small to rule out a possible interaction between the two interventions. Since intravenous rifampicin is an expensive and inconvenient route of administration, especially in low- and middle-income countries, two further oral rifampicin dose-finding trials were undertaken. It was found that a double and triple dose of oral rifampicin led to three and five-fold higher geometric mean total exposures in plasma, with proportional increases in CSF concentrations and without an increase in the incidence of grade 3–4 adverse events ^27, 37^.

**Figure 2.**
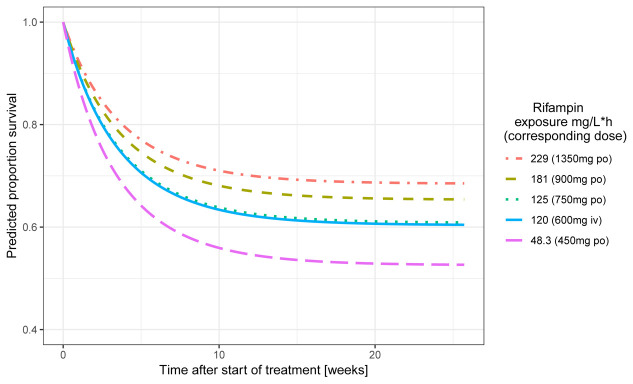
Influence of exposure to rifampicin in plasma on survival in Indonesian patients with TBM.

The PK and efficacy data from these three small trials were combined and related to each other in a recent combined analysis using population PK approach. The PK analysis included 133 individuals and 1150 rifampicin concentrations (170 from CSF) and the survival analysis included 148 individuals of whom 58 died and 15 dropped out. Higher individual plasma rifampicin exposures (AUC _0–24h_), lower age and higher baseline Glasgow coma scale (GCS) scores reduced the hazard of death ^38^. Figure 2 shows how simulations predicted an increase in 6-month survival from approximately 50% to approximately 70% upon increasing the oral rifampicin dose from 10 to 30 mg/kg, and predicted that even higher doses would further improve survival ^38^. Based on this analysis it was concluded that higher rifampicin exposures early during treatment substantially decrease the risk of death and that optimal dose of rifampicin in treatment of TBM should be further investigated in phase III trials.

In contrast, in a large randomised, double-blinded, placebo-controlled trial in Vietnam, adults with TBM received an intensified TBM regimen containing both 15 mg/kg/day oral rifampicin and levofloxacin plus standard isoniazid, pyrazinamide and ethambutol or standard of care TBM treatment for the first two months of treatment, there was no significant difference in 9-month mortality between the two groups (HR 0.94; 95% CI, 0.73 to 1.22; P = 0.66), though there was a survival benefit in those with isoniazid monoresistance (HR 0.34; 95% CI 0.15 to 0.76; P = 0.01) ^39, 40^.

The lack of overall effect in the Vietnam trial could be because the oral rifampicin dose was too low. The nested PK/PD study in 237 trial participants was published earlier this year and aimed to define exposure-response relationships in study participants. Rifampin 15mg/kg increased plasma and CSF exposures compared to 10mg/kg: day 14 plasma AUC _0–24_ increased from 48.2h•mg/L (range 18.2–93.8) to 82.5h•mg/L (range 8.7–161.0) and CSF AUC _0–24_ from 3.5h•mg/L (range 1.2–9.6) to 6.0h•mg/L (range 0.7–15.1). However, within the exposure range achieved, no relationship between rifampin exposure and survival was seen within the trial ^41^. It is possible, as seen in the PTB model ^34^ and in TBM PK-PD model ^38^, a rifampicin dose of greater than 15mg/kg is required to achieve exposures capable of reducing time to culture conversion and improving survival, which may explain the lack of exposure-response relationship seen in this trial. 

Alternatively, the reduction in mortality seen in the Indonesia trial may represent a type-I error due to a small sample size. In addition, Indonesian patients had more severe neurological dysfunction, with only 7% classified as having grade 1 disease severity according to the British Medical Research Council (BMRC) ^42^ TBM criteria at baseline, compared to 39% in Vietnam. These conflicting trials create equipoise as to whether higher dose rifampicin is beneficial or not; a question that HARVEST will be able to answer.

## Protocol

### 1. Hypothesis

Our primary hypothesis is that, among those with TBM, high dose oral rifampicin dosed at ~35 mg/kg/day will increase plasma, CSF and brain exposure to rifampicin, resulting in more rapid mycobacterial clearance, and improved clinical outcomes including: 1) survival, 2) more rapid resolution of coma, 3) improvement in functional status.

### 2. Main study objectives

***2.1 Primary objective.*** Our primary objective is to determine if high dose rifampicin, delivered orally at ~35 mg/kg/day for 8 weeks, is safe and improves 6-month survival compared to standard of care (rifampicin 10 mg/kg/day) for patients with TBM.

***2.2 Secondary objectives.*** Our secondary objectives are six-fold. We will compare the high dose rifampicin to standard of care for:

i. 12-month survival
ii. neurological disability and functional outcomes
iii. safety and tolerability
iv. hospital outcomes related to TBM
v. subsequent neurological deterioration
vi. management (incidence and outcomes) of drug induced liver injury (DILI)

### 3. Ancillary studies

To maximise research outputs from the trial, several ancillary studies are anticipated. These include:

***3.1 Clinical pharmacology studies.*** The objectives will be to characterise the PK of rifampicin in plasma and CSF between study arms, assess predictors of exposure to rifampicin in plasma and CSF, assess the relationship between rifampicin concentration and survival and evaluate the impact of high dose rifampicin on co-administered antiretroviral therapy (ART).

***3.2 Management of DILI.*** We hypothesise that the current guidelines for management of DILI in those with TBM result in the premature cessation of rifampicin and isoniazid, both critical in early therapy, placing patients at unnecessary risk of death and disability.

***3.3 Cost effectiveness analysis of the intervention.*** We will perform and economic evaluation of the cost effectiveness of the intensified TB treatment intervention from the perspective of the health payer in South Africa (middle income country), Indonesia (middle income country) and Uganda (low income country).

***3.4 Archiving blood samples for planned and future studies.*** We will store CSF, plasma, blood, and urine for studies related to:

1)   TBM diagnosis. This may include diagnostic testing for mycobacterial DNA, RNA, or proteins (e.g. Xpert MTB/RIF Ultra, next-generation sequencing, lipoarabinomannan (LAM), etc.)

Where consent has been given for host genomic studies we will store CSF, plasma, and blood for metagenomic (i.e. DNA and RNA) gene sequencing to compare gene expression related to:

2)   TBM prognosis (survival vs. death)

3)   Neurocognitive outcome

### 4. Design and setting

**Figure 3.**
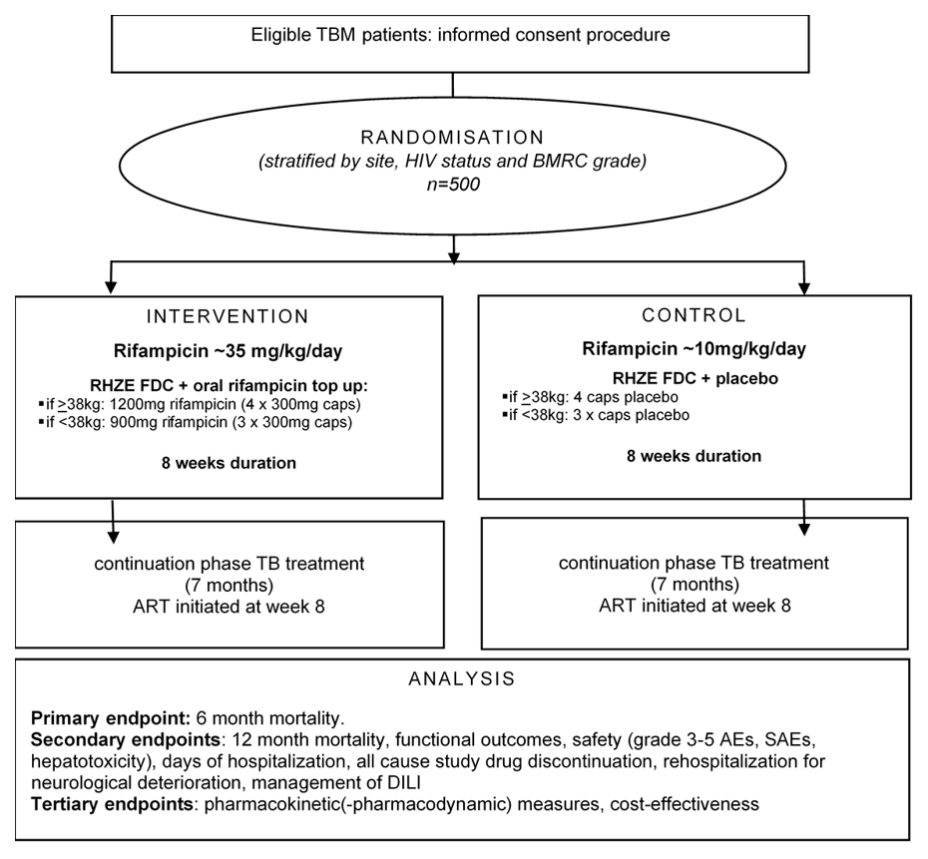
Schematic of study design. Abbreviations: TBM, tuberculous meningitis; BMRC, British Medical Research Council; R, rifampicin; H, isoniazid; Z, pyrazinamide; E, ethambutol; FDC, fixed dose combination; caps, capsules; ART, antiretroviral therapy; AE, adverse event; SAE, serious adverse event; DILI, drug-induced liver injury.

HARVEST is a parallel group, randomised, placebo-controlled, double blind, phase III multicentre clinical trial evaluating whether high dose oral rifampicin (~35 mg/kg/day) administered during the first 8 weeks of TBM treatment compared to standard of care (rifampicin ~10 mg/kg/day) improves outcome in TBM ( Figure 3). All participants will receive standard isoniazid (~5 mg/kg/day), pyrazinamide (~25 mg/kg/day), ethambutol (~20 mg/kg/day) plus corticosteroids.

The trial will be set in 5 sites across 3 countries: Hasan Sadikin Hospital, Bandung and Dr. Cipto Mangunkusumo Hospital, Jakarta in Indonesia; Mulago National Tertiary Referral Hospital, Kampala and Mbarara Regional Referral Hospital in Uganda; and Prince Mshiyeni Memorial Hospital, Durban in South Africa. 

### 5. Endpoints

***5.1 Primary endpoint.***

*1. 6-month survival*

This time point has been chosen since 95% mortality occurs by 6-months, and we anticipate our intervention will have the largest impact early in treatment. Mortality will be determined by active patient follow-up. Patients lost to follow-up during the first 8 weeks will be considered as failures and will count towards the primary endpoint. Participants lost to follow-up after 8 weeks will be censored.

***5.2 Secondary endpoints***

*1. 12-month survival*

*2. Functional and neurocognitive outcomes*

a) In patients presenting with altered mental status and depressed consciousness we will assess the number of days from randomisation until GCS 15 is achieved for ≥2 consecutive days.
b) We will assess functional outcomes by Liverpool Outcome Score at month 6 ( Table 2)
c) We will record quantitative neurocognitive performance Z-scores (QNPZ-8) derived from a test battery at 2 and 12 months (Uganda only). The test battery includes Grooved Pegboard test, Colour Trails 1 and 2 tests, WAIS-III Digit Symbol test, Finger Tapping test, WHO-UCLA Auditory Verbal Learning Test, Semantic Verbal Fluency test (category fluency) ^43^

**Table 2.** Liverpool outcome score ^44^.

5	Full recovery & normal neurological examination
4	Minor sequelae with mild effects on function or personality change or on medication
3	Moderate sequelae mildly affecting function, probably compatible with independent living
2	Severe sequelae, impairing function sufficient to make patient disabled
1	Death

*3. Safety and tolerability*

We will measure five safety and tolerability end points: i) clinical grade 3–4 adverse events (AEs) as classified by Division of AIDS (DAIDS) Toxicity Scale, ii) laboratory AEs grade 3–4, iii) serious adverse events, iv) DILI (alanine transaminase (ALT) or aspartate transaminase (AST) >3x upper limit of normal (ULN) with symptoms of hepatitis or >5x ULN without symptoms of hepatitis, v) discontinuation of TB treatment for >5 days in the first 8 weeks for any cause.

***5.3 Tertiary endpoints.*** Pharmacokinetic parameters and cost-effectiveness relate to the ancillary studies and will be reported separately from the main trial.

### 6. Inclusion and exclusion criteria

***6.1 Inclusion criteria.*** We will include adults (≥18 years old) who present with a first episode of TBM as clinically suspected by the attending physician (≥3 days of meningitis symptoms and CSF abnormalities) and TB treatment planned. Written informed consent must be obtained by the participant or surrogate in the case of altered mental state.

***6.2 Exclusion criteria.*** The participant will be excluded from the study if there is jaundice, known liver cirrhosis or elevated ALT >5x ULN. Due to dose adjustment of ethambutol and pyrazinamide required in renal failure, patients with an estimated glomerular filtration rate (eGFR) <30 ml/min will be excluded. Participants are ineligible if they have received more than five doses of any TB treatment within the previous seven days, if they have a known allergy to any standard TB drug or have known current or previous rifampicin-resistant *M. tuberculosis* infection. Participants should not be enrolled if they have additional active and confirmed CNS infection, have a contraindication to steroids, are unlikely or unable to attend regular clinic visits or are pregnant or breastfeeding. Due to interactions with rifampicin, HIV-infected individuals are ineligible if they require ongoing use of protease inhibitor-based antiretroviral therapy.

### 7. Randomisation and treatment allocation

Adults eligible for enrolment following a diagnostic screening lumbar puncture will be approached for enrolment consent. Randomisation will occur prior to administration of the sixth dose of TB treatment. A computer-generated permutated block randomisation algorithm of different sized blocks will randomise patients within a 1:1 ratio into the two trial arms.

Randomisation will be stratified by:

1. Clinical site
2. HIV-status
3. BMRC TBM disease grade I or II/III at time of randomisation

Equal randomisation will occur at each stratum. Randomisation schedules will be provided to each study site’s pharmacy in a listed sequence. Sequential, unique randomisation codes will be recorded on the study entry case report form (CRF) to assure no skipping of the randomisation order. All randomised participants will initiate their allocated study TB treatment within 48 hours of randomisation, and preferably on the same day.

The trial will be placebo-controlled and outcomes determined by study personnel who are all blinded to treatment allocation. Unblinding of individual patients will only occur in rare emergencies after approval from a Chief Investigator. Hepatotoxicity will not require unblinding as they will be managed according to the DILI sub-study algorithm, regardless of their randomisation arm.

***7.1 Treatment discontinuation.*** In order to prevent delay of urgent TBM therapy patients may be enrolled before baseline bloods are known to the study team. Study sponsored withdrawals may take place before day 15 for one of the following reasons

i. Baseline ALT >5xULN. Participants can be randomised before liver function tests (LFT) results are available to the study team, which may be up to 2 days in certain sites.
ii. Baseline eGFR <30 ml/min and fails to rise to ≥30 ml/min within 72 hours of enrolment. Pyrazinamide and ethambutol require renal dosing for long term use in those with an eGFR <30 ml/min which would complicate intervention and management.
iii. Rifampicin resistance identified after randomisation. Patients with isoniazid-mono-resistant TB may participate in the study.
iv. Confirmed CNS infection other than TBM, who have TBM treatment stopped prior to day 15.

### 8. Interventions

***8.1 Meningitis diagnostic tests.*** Standard of care meningitis testing involves an HIV test (if not already known to be HIV positive), a lumbar puncture with routine CSF analysis for white cell count and differential, protein, glucose and microscopy (e.g. Gram Stain, +/- Ziehl-Neelsen Stain, +/- India ink, cryptococcal antigen (HIV-infected participants), TB testing with GeneXpert MTB/Rif Ultra, CSF TB culture ± urine TB LAM. Whether separate screening consent or lumbar puncture consent is required will depend on local practice.

***8.2 Antituberculous therapy.*** HRZE fixed dose combination TB treatment consists of rifampicin (R) ~10 mg/kg/day, isoniazid (H) ~5 mg/kg/day, pyrazinamide (Z) ~25 mg/kg/day and ethambutol (E) 20 mg/kg/day. The intervention arm will consist of HRZE fixed dose combination tablets based on weight with additional ‘top up’ of four rifampicin 300mg capsules administered for the first eight weeks (unless <38 kg, in which case only three ‘top up’ rifampicin capsules are given). In the control arm, three or four additional placebo tablets will be given according to weight ( Table 3). After enrolment, participants will receive study TB treatment according to random treatment allocation as soon as possible, within 24 hours of enrolment. After the 8-week intervention period, both arms will be given rifampicin and isoniazid according to standard doses during the continuation phase of treatment for 7 to 10 months according to national guidelines. Medication will be given orally under directly observed therapy during hospitalisation, unless the patient cannot swallow, in which case tablets will be dispersed in water and administered via a nasogastric tube. 

**Table 3.** Administration of study drug.

Weight	Standard FDC RHZE (150/75/400/275 mg)	Additional Rifampicin 300mg caps / placebo	Total R dose in intervention arm	Total R dose in placebo arm	Dose of other TB drugs (H/Z/E)
30–37 kg	2 tabs	3	1200mg	300mg	150/800/550
38–54 kg	3 tabs	4	1650mg	450mg	225/1200/825
55–70 kg	4 tabs		1800mg	600mg	300/1600/1100
≥ 71 kg	5 tabs		1950mg	750mg	375/2000/1375

R = rifampicin, H = isoniazid, Z = pyrazinamide, E = ethambutol.

***8.3 Antiretroviral therapy.*** HIV therapy will be provided in partnership with local HIV services. HIV-infected participants not already receiving ART will be recommended to initiate ART at 8-weeks following TB treatment initiation, according to international guidelines ^45^. All HIV-infected participants will receive cotrimoxazole prophylaxis, unless allergic, according to national guidelines.

***8.4 Adjunctive corticosteroid treatment.*** A meta-analysis found that adjunctive corticosteroid treatment improve survival among HIV-uninfected persons, and as such is recommended for all patients with CNS TB according to WHO guidelines ^46^. The benefit in HIV-infected persons is less clear and may be answered by the ACT-HIV trial currently underway in Vietnam and Indonesia ( NCT03092817), with results expected in 2020. Corticosteroid administration will be standardised for all participants as per the trial’s standard operation procedures (SOP), though clinical discretion can be used where medical complications arise.

***8.5 Blood test monitoring.*** Routine blood test monitoring will occur on day 1, 3, 7, 14, week 4 and week 8 to monitor for rifampicin toxicity. See Table 4 and Table 5.

**Table 4.** Schedule of events during hospitalisation.

Study item	Screening	Enrolment Day 1	Day 2	Day 3	Day 7	Day 14 ^*^	Weekly till hospital discharge	Hospital discharge	Further Hospitalisation
Visit window (days)					+3	+3	+3		
Screening Consent (Uganda only)	X								
Assess eligibility criteria		X							
Patient information and enrolment consent		X							
Clinical history and examination									
Past medical history	X	X							
Medication review	X	X	X	X	X	X	X	X	X
Document HIV status		X							
Current symptoms	X	X	X	X	X	X	X	X	X
Physical examination	X	X	X	X	X	X	X		X
GCS score ^a^	X	X	X	X	X	X	X	X	X
BMRC disease grade		X							
Adverse event assessment		X	X	X	X	X	X	X	X
Investigations									
HIV-test (if not known positive)	X								
Cryptococcal antigen if HIV+ (blood)	X								
Sodium	X ^c^	X ^g^			X				X
Potassium		X							
Glucose (bedside)	X ^c^								
Creatinine ^d^	X ^c^	X ^g^			X				X
Hepatic panel ^e^	X ^c^	X ^g^			X	X			X
Blood Count (including differential)	X ^c^	X ^g^			X				X
CD4 if HIV-positive	X ^c^	X ^g^							
Pregnancy test	Women ^c^								
Chest radiograph	+/- ^c^								
Urine sample +/- storage ^f^	X ^c^								
Lumbar puncture for Xpert, culture and storage after enrolment and if not contra- indicated (SA) ^h^		X							
PK/PD sub-study in participating sites: sparse PK sampling ^g^ (plasma x3 and CSF x1 sample)			X						
Blood / DNA / RNA storage		X	X (if RIF PK)			X (if ART PK, or at hos d/c)			Optional (with consent)

Footnotes: *week 2 visit may be performed as an inpatient or outpatient depending on the time of hospital discharge ^a^ GCS will be captured daily during hospitalisation on a log by study team or routine care providers ^b^ Adverse events will be recorded according to DAIDS toxicity scale ^c^ Provided as standard of care in some of the trial sites. Where not performed as part of SOC the test will be study sponsored. ^d^ Additional renal monitoring will be undertaken in those with abnormal baseline creatinine ^e^ Hepatic panel = alanine aminotransferase (ALT), alkaline phosphatase (ALP) and total bilirubin. Hepatitis BsAg, Hepatitis C Ab will be added if baseline ALT is elevated. In the event of DILI hepatic panel will be performed as per DILI SOP. ^f^ Urine sample may be collected from HIV-positive patients during screening for testing with TB-LAM (lipoarobinmannan) as part of TB work-up ^g^ Baseline bloods must occur at either during screening or at enrolment visit. It is possible these visits will be on the same day. If baseline bloods were done at screening and enrolment occurs >72 hours later baseline blood tests will be repeated. ^h^ LP may be performed to do initial Xpert/TB culture tests (if not done through routine care) or to repeat these tests to improve diagnostic yield of TBM. Investigations requested by the treating physician as part of routine care (e.g. exclusion of additional causes of meningitis as appropriate) may also be performed on CSF obtained at this timepoint GCS = Glasgow come scale, BMRC = British Medical Research Council, PK = pharmacokinetic, PD = pharmacodynamic.

**Table 5.** Outpatient Schedule of events.

Study item	Wk 2 ^*^	Wk 4	Wk 8	Wk 12	Wk 18	Wk 24	Wk 36	Wk 52	Sick Visit
Visit window (weeks)	(1,3)	(3,6)	(6, 10)	(10, 13)	(13, 21)	(21, 30)	(30, 44)	(44, 60)	As needed
Dispensing of study drug	X	X	X	standard fixed dose therapy for 7–10 months as per local guidelines					
Interim history		X	X	X	X	X	X	X	X
AE assessment		X	X	X	X	X	X	X	X
Medication review	X	X	X	X	X	X	X	X	X
Adherence assessment	X	X	X	X	X	X	X	X	X
Physical Examination	X	X	X	X		X			X
Liverpool Outcome Score						X			
Detailed neurocognitive follow-up (Uganda)			X					X	
Sodium									± if clinically indicated
Creatinine									± if clinically indicated
Liver Panel ^a^	X	X	X						± if clinically indicated
Full blood count, differential									± if clinically indicated
CSF analysis & storage									± if clinically indicated
Initiate or switch ART ^b^			X						
Storage of blood (ml)		X (if ART PK)				X (optional, HIV-pos)	X (optional, HIV-pos)		± if clinically indicated

Footnotes: *week 2 visit may be performed as an inpatient or outpatient depending on the time of hospital discharge All visits should ideally take place in person but can, in certain circumstances, be done by telephone or via home visit (if patient consents) ^a^Liver panel = ALT; alanine aminotransferase, ALP; alkaline phosphatase, total bilirubin ^b^ HIV-infected patients not receiving effective ART. ART initiation via local HIV service and in-line with ART management SOP. AE = adverse event, CSF = cerebrospinal fluid, ART = antiretroviral therapy

***8.6 Pharmacokinetic sampling.*** In Indonesian and Ugandan sites intensive pharmacokinetic sampling will take place on day 2, with plasma samples collected at 3 time points, as well as a single CSF sample. These sites have experience in prior PK studies.

***8.7 Neurocognitive testing.*** In Kampala detailed neurocognitive assessment will take place at 2 and 12 months as there is a nurse trained and experience in neurocognitive assessment at that site.

### 9. Adverse events and safety reporting

The definitions of the EU Directive 2001/20/EC Article 2 based on the principles of the international committee of harmonisation good clinical practice (ICH GCP) apply to this trial protocol. The investigational medicinal product (IMP) is high dose rifampicin and the comparator is standard dose rifampicin. All adverse events will be assessed for seriousness, causality and expectedness. Causality in relation to the IMP is assessed as unrelated, unlikely, possible, probably or definite based on temporal relationship and clinical judgement. If the event is serious and unrelated or unlikely to be related it is classified as a serious adverse event (SAE). If the event is classified as possible, probably or definitely related it is classified as a serious adverse reaction (SAR). Expectedness of the adverse reaction is assessed using the summary of product characteristics (SPC). An unexpected adverse reaction is one not previously reported, or more severe or frequently reported in the SPC. If an SAR is assessed as unexpected it becomes a suspected unexpected serious adverse reaction (SUSAR). Intensity will be assessed using the Division of AIDS (DAIDS) Table for Grading the Severity of Adult and Paediatric Adverse Events.

The coordinating centre (Infectious Disease Institute, Kampala, Uganda) will be notified of all grade 3–4 AEs, SAEs (including deaths) and SARs within 72 hours. Investigators will notify the coordinating centre of all SAEs occurring from the time of randomisation until 6 months (time of primary endpoint). SAEs, SARs, and SUSARs will be reported to the coordinating centre, local and international Institutional Review Boards (IRB) until trial closure, as required by regulatory guidelines. The coordinating centre will report SAEs, SARs and SUSARs to the regulatory authorities and research ethics committees. SAEs and SUSARs will be reported to regulatory authorities within 7 days of the coordinating centre becoming aware of the event.

Quarterly reports pooled over both arms on subject toxicity will be provided to the trial steering committee (TSC). The trial will be monitored by an external and independent data and safety monitoring board (DSMB). The independent data safety and monitoring board (DSMB) review data after 25%, 50% and 75% of participants have completed the 2-month visit, and will have full access to accumulating data on efficacy, safety and treatment group assignment. A Lan-DeMets spending function analogue of the O’Brien-Fleming boundaries will be provided for the 6-month survival outcome with each DSMB report. Early termination or protocol modification will be considered if the O-Brien-Fleming boundary is crossed. If the trial steering committee, Sponsor, IRB or regulatory authorities request the DSMB can modify the frequency of interim analysis.

### 10. Data collection

***10.1 Baseline and subsequent assessment.*** Participants will be enrolled and hospitalised for at least seven days, discharged and followed up on a regular schedule until week 52 ( Table 4, Table 5). Hospitalised patients will be reviewed by a study doctor daily on working days in the first week, then 2–3 times per week until hospital discharge. During weekends and public holidays, the patients can be reviewed by the medical team who will alert the study team of any clinical events. Telephone follow-up and home visits may be used to minimise inconvenience to physically disabled participants. If clinical deterioration occurs participants will be seen at unscheduled visits. Additional laboratory and radiological tests can be undertaken at the discretion of the physician with agreement of the site principal investigator (PI).

***10.2 Data handling and data management.*** Source documents include detailed CRFs, laboratory and radiology reports, pharmacy dispensing records and external medical records. Data entry will occur via the DataFax system, whereby paper-based CRFs are scanned, emailed to a server and data entered by intelligent character recognition. After initial automated error checking, secondary review for accuracy will be performed by the DataFax team at the Infectious Diseases Institute, Uganda. The DataFax system allows for automated data queries to alert for any missing data on an ongoing basis. This also allows for permanent archiving and potential remote review by oversight bodies. Study specific forms will be harmonised between all study sites enabling multi-site data management. The investigator will retain essential study documents completion of the study, as per local guidelines. Digital images of the source documents will be retained for an indefinite period.

***10.3 Quality control and assurance.*** Internal and external site monitoring will be conducted to ensure that the human subject protection, study procedures, laboratory, study intervention administration, and data collection processes are of high quality and meet the sponsor, ICH E6 and regulatory guidelines. The study may be subject to audit by the Infection Diseases Institute, Uganda under their remit as sponsor, as well as other regulatory bodies to ensure adherence to GCP.

### 11. Statistical considerations

***11.1 Sample size.*** The target sample size is 500 subjects. The randomization will be stratified by site, BMRC TBM severity grade (I vs II/III) and HIV-status. The goal for completion of enrolment is <30 months based on an accrual of ~200 subjects per year. The primary analysis will be intention to treat. Type 1 error is 0.05 (2-sided) and power = 0.80. We estimate a ~50% cumulative death rate with standard TBM treatment. Experience in recent studies includes an 8-week mortality of 43% (51/120) in TBM patients (88% HIV-infected) in Cape Town ^12^. Ruslami *et al.* reported 8-week mortality of 55% in TBM patients who received standard-of-care TB treatment in the Indonesian trial, however, 88% of patients were HIV-uninfected ^27^. Two large RCTs have further reported mortality of 40–45% ^3, 45^ in HIV-associated TBM in Vietnam, but with further experience and enhanced excellence in care reduced mortality to 27% in their most recent 2016 published trial (at the perhaps most experienced TBM site in any low and middle-income country) ^39^. The potential effect size is based on a conservative effect of at least 10% absolute improvement in all-cause 8-week mortality, which would be sufficient to change clinical practice. In the prior Ruslami *et al.* trial in Indonesia (n=60), there was a 56% relative risk reduction in 8-week mortality with 24% (7/29) mortality using IV rifampicin vs. 55% (17/31) mortality using oral rifampicin therapy ^27^. This 31% absolute reduction in mortality may be over-estimated based on the small sample size with a 95% CI of 7.3% to 54% mortality reduction. Both study-sponsored withdrawals and loss to follow-up are each expected to be no more than 5%.

A two-sided log rank time-to-event analysis with an overall sample size of 500 subjects (250 in the standard of care arm and 250 in the high-dose rifampicin arm) achieves 80% power at a 0.05 significance level to detect a hazard ratio of 0.68 when the proportion surviving in the control group is 50%, assuming up to 5% lost-to-follow up and no more than 5% study-sponsored withdrawals. This equates to a 13% absolute improvement in survival. If lost to follow up is less, then power increases slightly. Prior lost to follow up rate has been <1% in recent meningitis trials. The 12-month follow-up period makes lost-to-follow-up more likely, thus we have used an assumption of 5%. For survival analyses, participants who are lost-to-follow-up before 8 weeks will be counted as failures, and participants lost after 8 weeks censored.

***11.2 Primary analysis.*** The primary analysis will be by intention to treat, comparing 6-month survival between the high dose rifampicin arm to the standard of care arm. Time-to-event methods, including Kaplan-Meier cumulative event curves with log-rank tests and proportional hazards regression models will be used to summarize excess risk of death. The primary analysis will be an unadjusted proportional hazards regression model. We will assess the assumption of proportionality of the hazards over time, to investigate if there is any evidence of an early difference that reverses with additional follow-up. Several pre-specified sensitivity analyses will also be performed: a model stratified by the randomization strata (clinical site, HIV status and BMRC TBM grade), and another model adjusted for differences in baseline covariates.

***11.3 Secondary analysis.*** Time-to-event methods will also be used for the secondary events of 12-month survival, adverse events and re-hospitalisation due to neurological deterioration. Logistic regression models will be used to compare the treatment arms for binary outcomes (including normalization of mental status and all-cause drug discontinuation of more than 5 days), or Fisher’s Exact tests if appropriate. Ordinal logistic regression models will be used to compare the treatment arms for the Liverpool Outcome Score. General linear models or Wilcoxon rank-sum tests will be used to compare the treatment arms for differences in continuous-valued measurements (including QNPZ-8). 

***11.4 Planned subgroup analysis.*** Proportional hazards regression models for 6-month survival will also be performed for a priori subgroups of interest:

- Glasgow Coma Score at presentation
- TBM Diagnostic certainty (Definite/Probable versus Possible) ^4^
- BMRC TBM disease severity ^3^
- HIV status
- ART status at study entry
- Infection with isoniazid mono-resistant *Mycobacterium tuberculosis* strains
- CSF and plasma rifampicin exposures

***11.5 Analysis of ancillary studies.*** The results of ancillary studies will be reported separately from the main trial. Findings of the diagnostic sub-study will be reported in line with the Standards for the Reporting of Diagnostic Accuracy studies (STARD) guidelines.

### 12. Ethical considerations

***12.1 Confidentiality.*** All participant-related information (including CRFs, laboratory specimens, evaluation forms, reports, etc.) will be kept strictly confidential. All records will be kept in a secure, locked location and only research staff will have access to the records. Participants will be identified only by means of a coded number specific to each participant. HIV and TB clinic records will be kept in the local HIV and TB clinics as per local practice.

All computerized databases will identify participants by numeric codes only, and will be password-protected. Upon request, participant records will be made available to the study sponsor, the sponsor’s monitoring representative, representatives of the sponsor and applicable local and national regulatory entities.

***12.2 Consent.*** All informed consent documentation will be read in full to potential study participants or their legally acceptable surrogate i.e. caregiver or next of kin. Consent will be obtained with an approved consent form in English or the local language. The potential participant or surrogate will be given sufficient time to review, consider and discuss potential questions. Participants are still eligible for enrolment having received up to 5 days of TB therapy, thus study window for eligibility is not a coercive influence. We estimate 50% of the study population will have altered mental status at initial hospital presentation, therefore may have surrogate consent provided by proxy from their caregiver or next of kin.

Upon restoration of normal mental status, subjects enrolled with proxy consent will be re-consented. We will request permission from the ethics committees to use data of participants who do not regain ability or die prior to providing consent themselves. A person who speaks and understands the language of the informed consent document but does not read and write can be enrolled in a study by “making their mark” via a thumbprint on the informed consent document. The entire consent process and thumbprint will be witnessed by an impartial, literate third party. The witness’s name, signature and relationship will be recorded on the informed consent document.

***12.3 Sample use and storage.*** An optional additional storage consent will be obtained for long-term storage of blood, CSF and urine for the purposes of future research at certain sites based on capacity. Absence of this consent does not affect eligibility for enrolment or study procedures and storage requests required by this protocol.

***12.4 Withdrawals.*** Subjects may withdraw consent at any time during the study. Study-sponsored withdrawals may be done prior to study day 15 (section 7.1). If subjects are withdrawn from the study, for whatever reason, they will be eligible to continue to receive TB treatment from a primary TB clinic of their choice. Patients enrolled in the study who choose to leave hospital early against medical advice may continue to participate in the study if they wish. Additional phone calls by study personnel will encourage the subject to seek follow up TB care and to re-join the trial per the ongoing schedule of events. For persons with study-sponsored withdrawal because of an alternative brain infection or pathology other than TBM, referral will be made to appropriate clinical service. Participants will be asked if they would like their accrued data to be destroyed.

***12.5 Ethical approval.*** The study coordination centre has obtained approval from Mulago Hospital Institutional Review Board (MHREC 1554) and the Uganda National Council of Science and Technology (HS428ES). In South Africa, approval will be obtained from the Biomedical Research Ethics Committee of the University of KwaZulu-Natal, the Provincial Department of Health of KwaZulu-Natal and the South African Health Products Regulatory Authority. In Bandung, approval will be obtained from Research Ethics Committee of Universitas Padjadjaran Bandung, and in Jakarta, approval will be obtained from the Research Ethics Committee of the Faculty of Medicine Universitas Indonesia and Oxford Tropical Research Ethics Committee. Likewise, any future amendments to the study protocol will be approved by the trial steering committee and then approved by site(s)’ IRB and other regulatory bodies as required before being implemented. 

***Trial registration.*** The study was registered on the ISRCTN registry on 17 July 2019 under the registration number ISRCTN15668391.

### 13. Trial committees

The trial sponsor is the Infectious Diseases Institute, Uganda. The trial management group (TMG) will oversee day-to-day management of the trial and is formed of the chief investigators, site PIs in Uganda, South Africa, Indonesia, a neurologist and statistical advisor. The TMG will meet weekly. The TSC has members of the TMG and independent members. The TSC provides supervision for the trial and advice through the independent Chair. The DSMB will advise the TSC regarding continuation, modification or premature closure of the trail. The DSMB is independent from the sponsor.

### 14. Dissemination of findings

We will share results though presentations at scientific conferences and in peer-reviewed open-access journals. Electronic data will be stored on the DataFax server housed at the US National Institute of Health. Anonymised data can be accessed on written request to the data manager after approval by the Sponsor and investigators.

### Study status

The trial is currently seeking national drug regulatory authority approvals.

## Discussion

TBM continues to have unacceptably high morbidity and mortality despite current WHO recommended therapy. High dose rifampicin is safe, tolerable and has favourable CNS penetration compared to standard doses. While survival benefit has been demonstrated, multiple studies in different populations with varying interventions have yielded inconsistent results. In addition to Harvest, a further factorial design phase III RCT (Intense TBM, NCT04145258) exploring high dose rifampicin (35mg/kg) with or without adjunctive linezolid and aspirin is planned in Madagascar, Uganda, South Africa and Côte d’Ivoire. These complementary phase III randomised, placebo-controlled, double-blinded, multi-centre trial will definitively answer the question of whether high dose rifampicin improves survival in TBM.

## Data availability

### Underlying data

No data are associated with this article
