# Supplementary material for: High dose oral rifampicin to improve survival from adult tuberculous meningitis: A randomised placebo-controlled double-blinded phase III trial (the HARVEST study)
Source: Wellcome Open Res. 2020 Aug 25;4:190. Originally published 2019 Dec 2. [Version 2] doi: 10.12688/wellcomeopenres.15565.2 (PMC7542255; doi:10.12688/wellcomeopenres.15565.2)
Supplement: Peer review history

# Peer review: Reviewer response for version 2

**DOI:** 10.21956/wellcomeopenres.17819.r40134

**Published:** 2020-10-07

**Authors:** Olivier Marcy

**Affiliations:** 1 Centre INSERM U1219, Bordeaux Population Health, University of Bordeaux, Bordeaux, France

I have reviewed the revised version of the manuscript by Marais, Cresswell *et al*. on their high dose rifampin trial to reduce mortality in patients with TB meningitis. Authors have revised the protocol and answered all my comments appropriately. The results of this trial as well as other trials in the field of intensified TBM treatment are much awaited by the scientific community, public health decision makers, clinicians and the patients suffering from this deadly disease.

Is the study design appropriate for the research question?

Yes

Is the rationale for, and objectives of, the study clearly described?

Yes

Are sufficient details of the methods provided to allow replication by others?

Yes

Are the datasets clearly presented in a useable and accessible format?

Not applicable

Reviewer Expertise:

TB-HIV co-infection in children and adults, TB meningitis, TB diagnosis in children.

I confirm that I have read this submission and believe that I have an appropriate level of expertise to confirm that it is of an acceptable scientific standard.

# Peer review: Reviewer response for version 1

**DOI:** 10.21956/wellcomeopenres.17046.r39145

**Published:** 2020-07-02

**Authors:** Tihana Bicanic

**Affiliations:** 1 Centre for Global Health, Institute for Infection and Immunity, St George's, University of London, London, UK; 2 St George's Hospital NHS Trust, London, UK

Marais, Cresswell and collegues present an important, well written, comprehensive trial protocol for a phase III multi-site RCT of high vs standard dose rifampicin in the treatment of TBM in HIV-infected and uninfected patients in Africa and Asia.

 The rationale for the study is based on sound phase 2 data, however (as mentioned by Reviewer 1) I would urge the authors/investigators to expand a little on the rationale for the choice of the 35mg/kg rifampicin dose.

 The study design, study endpoints and sample size calculation are sound.

 I have a few minor suggestions for improvement of the manuscript/protocol:

1. Introduction - limitations of standard dose rifampicin - are there any data (animal or human) on brain (in addition to CSF) penetration of rifampicin? If so, include.
2. Introduction - fig 1- this figure seems quite generic and out of context and could be omitted.
3. High dose rifampicin justification: these are key background data to the dosing chosen in the trial - which is not sufficiently justified here - suggest expand and/or perhaps include diagram summarising any preliminary data from animal/human phase II studies - could replace fig 1.
  Specifically need to expand any known data on relationship between dose and mycobacterial clearance - is there a threshold? I realise no CFU data in TBM specifically - but from animal pulmonary TB models/human EBA data is there a correlation between higher doses rif and CFU counts/faster clearance. You mention time to culture conversion - important to cite the actual data for all experimental arms in PanACEA.
4. Safety of rifampicin: 'It was found that a three-fold increase in oral rifampicin was safe, and resulted in a large increase is CSF and plasma exposure' - please be more specific - to what dose, and what was size of increase?
  You mention PK data from Vietnam trial awaited - the trial was published in 2016/17- are these data now available to you?
5. Ancillary studies - management of DILI - it is unclear how management of DILI will be assessed as an endpoint/compared between groups (agree with Reviewer 1 comments).
6. Inclusion criteria, under case definition -' CSF abnormalities' very non-specific? Suggestive of meningitis? We all realise a lot of treatment is empiric - if clinician judgement only then state so. Agree with reviewer 1 point about which diagnostics likely to be available to clinicians at each site upon which to base inclusion (and subsequent exclusion is alternative diagnosis found).

Is the study design appropriate for the research question?

Yes

Is the rationale for, and objectives of, the study clearly described?

Yes

Are sufficient details of the methods provided to allow replication by others?

Yes

Are the datasets clearly presented in a useable and accessible format?

Not applicable

Reviewer Expertise:

Infectious diseases Phase III clinical trial design and conduct in LMICs; microbial clearance phase II studies in cryptococcal meningitis.

I confirm that I have read this submission and believe that I have an appropriate level of expertise to confirm that it is of an acceptable scientific standard.

# Response

**Published:** 2020-08-03

**Authors:** Fiona Cresswell

**Affiliations:** London School of Hygiene & Tropical Medicine, UK

We thank Dr Bicanic for her expert comments on the protocol and address them in turn below.

Comment 1. The rationale for the study is based on sound phase 2 data, however (as mentioned by Reviewer 1) I would urge the authors/investigators to expand a little on the rationale for the choice of the 35mg/kg rifampicin dose.

*Response 1. We have added further detail explaining the rationale for this choice of dose in pulmonary TB treatment, as shown below. We have also added further detail to the description of the PK-PD model-based analysis using data from Indonesian patients with TBM. Together, we hope this makes the rationale for this choice of 35mg/kg dose in the Harvest trial clear.*

*"The PanACEA multi-arm multistage trial showed that time to stable culture conversion in liquid media was faster in the 35 mg/kg rifampicin group than in the control group (median 48 days vs 62 days, adjusted hazard ratio 1·78; 95% confidence interval (CI) 1·22–2·58, p=0·003), but not in other experimental arms. In an additional analysis, increasing rifampicin exposure was also associated with shortened time to stable culture conversion. The effect did not plateau, indicating that doses >35 mg/kg could be yet more effective.*

*High-doses rifampicin was also associated with a greater estimated fall in M. tuberculosis bacillary load in sputum of patients with pulmonary TB at 2 weeks follow up, i.e. high-dose rifampicin showed increased ‘early bactericidal activity’. Recent analyses of one of these latter trials (the PanACEA HIGHRIF1 trial) included doses up to 40 mg/kg, demonstrating a further effect of rifampicin exposure on early bactericidal activity and clinical trial simulations showed greater early bactericidal activity for 50 mg/kg rifampicin. Recent evaluations of administration of this 50 mg/kg rifampicin dose show that this dose is not well-tolerated. The maximum tolerable dose for rifampicin in PTB is therefore set at 40 mg/kg daily (R. Aarnoutse and L. te Brake, personal communication, data from the PanACEA HIGHRIF1 trial).*

*In summary, data from pulmonary TB have shown that higher rifampicin doses, starting with at least 20 mg/kg daily but certainly at 35 mg/kg orally, result in strongly increased systemic exposures to rifampicin, were safe, tolerated, and resulted in increased response, as reflected in improved early bactericidal activity and shorter time to culture conversion. These lessons learnt in pulmonary TB are relevant to TBM treatment, considering the key role of rifampicin in TBM treatment and the limited CSF penetration seen at standard dose.*

*Further, model-based meta-analysis of data from TB meningitis patients in Indonesia predicted an increase in 6-month survival from approximately 50% to approximately 70% upon increasing the oral rifampicin dose from 10 to 30 mg/kg, and predicted that even higher doses would further improve survival”*

Comment 2. The study design, study endpoints and sample size calculation are sound.

I have a few minor suggestions for improvement of the manuscript/protocol: 

Comment 3. Introduction - limitations of standard dose rifampicin - are there any data (animal or human) on brain (in addition to CSF) penetration of rifampicin? If so, include.

*Response 3. Thank you for raising this point. There is some interesting recent data on brain (intralesional) rifampicin pharmacokinetics derived from rabbit models of experimentally induced TBM using serial noninvasive dynamic 11C-rifampin positron emission tomography (PET) over 6 weeks. Rifampin penetration into infected brain lesions is limited, spatially heterogeneous, and decreases rapidly as early as 2 weeks into treatment. Moreover, rifampin concentrations in the cerebrospinal fluid did not correlate well with those in the brain lesions. First-in-human 11C-rifampin PET performed in a patient with TBM confirmed these findings. PK modeling using this data predicted that rifampin doses (≥30 mg/kg) were required to achieve adequate intralesional concentrations in TBM (Tucker EW, Sci Transl Med, 2018). This information and citation have been added to the protocol.*

Comment 4. Introduction - fig 1- this figure seems quite generic and out of context and could be omitted.

*Response 4. We have decided to retain the figure as we are keen to visually highlight that fact that the CSF compartment, blood and brain compartment are distinct (yet related).*

Comment 5. High dose rifampicin justification: these are key background data to the dosing chosen in the trial - which is not sufficiently justified here - suggest expand and/or perhaps include diagram summarising any preliminary data from animal/human phase II studies - could replace fig 1. Specifically need to expand any known data on relationship between dose and mycobacterial clearance - is there a threshold? I realise no CFU data in TBM specifically - but from animal pulmonary TB models/human EBA data is there a correlation between higher doses rif and CFU counts/faster clearance. You mention time to culture conversion - important to cite the actual data for all experimental arms in PanACEA.In the absence of clearance data from CSF (animal nor human), we have added further detail relating to PK-PD targets from murine PTB and findings relating to clearance in human PTB from the PanACEA studies.

*Response 5. In plasma, the historical recommended target peak concentrations of rifampicin are 8-24mg/L. This historical target is based on rifampicin concentrations typically observed in healthy adults (i.e. the reference range after a dose of 10mg/kg orally), and is not optimized based on pharmacokinetic-pharmacodynamic (PK-PD) data. It is now accepted that total exposure to TB drugs, or the area under the 24-h concentration–time curve (AUC _0–24_), is more relevant to the efficacy of first-line TB drugs. Ideally, AUC _0–24 _is considered together with the minimum inhibitory concentration (MIC) of the mycobacteria to yield an AUC _0–24_/MIC ratio that is supposed to best predict response in murine models (Magis-Escurra, Int J Antimicrob Agents, 2014).*

**

*A large meta-analysis of rifampicin pharmacokinetic data from 70 studies with PTB patients found that the mean plasma rifampicin peak concentration at steady state is only 5.79mg/L (below the historical target concentration) and doses of >25mg/kg are needed to achieve the PK-PD target derived from murine studies (AUC/MIC >271) (Stott, J Antimicrob Chemother, 2018).*

**

*We have added more detail about the PanACEA studies. The PanACEA multi-arm multistage trial showed that time to stable culture conversion in liquid media was faster in the 35 mg/kg rifampicin group than in the control group (median 48 days vs 62 days, adjusted hazard ratio 1·78; 95% confidence interval (CI) 1·22–2·58, p=0·003), but not in other experimental arms. In an additional analysis, increasing rifampicin exposure was also associated with shortened time to stable culture conversion. The effect did not plateau, indicating that doses >35 mg/kg could be yet more effective (though are unlikely to be well tolerated). The exposure-response relationship for high-dose rifampicin has been explored by using pharmacokinetic-pharmacodynamic modelling based on data from the HIGHRIF studies. Rifampicin exposure was a significant covariate on the bacterial kill rate in sputum resulting in increased early bactericidal activity (Svensson R. J Infect Dis. 2018; 218(6):991-999).*

**

Comment 6. Safety of rifampicin: 'It was found that a three-fold increase in oral rifampicin was safe, and resulted in a large increase is CSF and plasma exposure' - please be more specific - to what dose, and what was size of increase?

You mention PK data from Vietnam trial awaited - the trial was published in 2016/17- are these data now available to you?

*Response 6. Since intravenous rifampicin is an expensive and inconvenient route of administration, especially in low- and middle-income countries, two further oral rifampicin dose-finding trials were undertaken. We have added further detail to the protocol paper reflecting the below results.*

**

*In the first follow-up study in 30 Indonesian TBM patients, exposures to and safety of higher oral rifampicin doses (750 mg, ca. 17 mg/kg orally; 900 mg, ca. 20 mg/kg orally) were compared with the same 600 mg IV rifampicin dose (Yunivita et al. Int J Antimicrob Agents. 2016;48:415-418), as studied by Ruslami et al. Both higher oral rifampicin doses resulted in approximately similar plasma AUC _0-24_ but lower plasma C _max_ values compared with 600 mg IV. In a second follow-up study, 60 Indonesian TBM patients were assigned in a random double-blinded study to standard 450 mg, 900 mg or 1350 mg (10, 20 and 30 mg/kg for Indonesian subjects) oral rifampicin combined with other TB drugs for 30 days [Dian S. Antimicrob Agents and Chemotherapy. 2018;62(12):e01004-12]. Double and triple dose of oral rifampicin led to three and five-fold higher geometric mean total exposures in plasma, with proportional increases in CSF concentrations, without an increase in incidence of grade 3/4 adverse events. Overall, tripling the standard dose was considered to be safe.*

**

*The PK-PD analysis from the 2016 Vietnamese intensified TB treatment trial were published earlier this year (Ding J, Clin Pharmacol Ther, 2020). The nested PK/PD study in 237 trial participants was published earlier this year and aimed to define exposure-response relationships in study participants. Rifampin 15mg/kg increased plasma and CSF exposures compared to 10mg/kg: day 14 plasma AUC0-24 increased from 48.2h·mg/L (range 18.2-93.8) to 82.5h·mg/L (range 8.7-161.0) and CSF AUC0-24 from 3.5h·mg/L (range 1.2-9.6) to 6.0h·mg/L (range 0.7-15.1). However, within the exposure range achieved, no relationship between rifampin exposure and survival was seen within the trial. It is possible, as seen in PTB studies (Svennson R), and in TBM PK-PD models (Svensson E), a rifampicin dose of greater than 15mg/kg is required to achieve exposures capable of reducing time to culture conversion and improving survival, which may explain the lack of exposure-response relationship seen in this study.*

Comment 7. Ancillary studies - management of DILI - it is unclear how management of DILI will be assessed as an endpoint/compared between groups (agree with Reviewer 1 comments).Thank you for seeking clarity on this.

*Response 7. We hope the response given to reviewer 1, and copied here, makes the purpose of this sub-study a little clearer. The aim of the sub-study is to determine the safety of a pragmatic management strategy, and does not involve comparison between trial arms. Based on safety data from prior Indonesian high dose rifampicin studies it appears that the majority (8 of 9) of TBM patients who develop transaminitis (ALT of <10x upper limit of normal, ULN), liver enzymes settle without interruption of anti-TB drugs. Where transaminitis occurs, we therefore plan to closely monitor liver function tests whilst continuing TB treatment unless ALT exceeds a threshold of 10x ULN, at which point we will follow an algorithm of stopping and reintroducing TB drugs sequentially. Under trial endpoint #6 we use “management of DILI” as a summary term under which we aim to describe incidence of DILI, frequency and duration of drug interruptions, hepatic outcomes, neurological outcomes. We recognise that “management of DILI” is a little non-specific so we have added “incidence and outcome” and will notify IRBs at the time of next regulatory amendments.*

Comment 8. Inclusion criteria, under case definition -' CSF abnormalities' very non-specific? Suggestive of meningitis? We all realise a lot of treatment is empiric - if clinician judgement only then states so. Agree with reviewer 1 point about which diagnostics likely to be available to clinicians at each site upon which to base inclusion (and subsequent exclusion is alternative diagnosis found). This is challenging point and something we discussed at length when writing the protocol. All sites will provide a minimum set of CSF tests which includes Gram’s stain, bacteriological culture, Xpert MTB/RIF Ultra, TB culture, Cryptococcal antigen if HIV-positive. Availability of other tests varies by site (including viral PCR, syphilis serology, fungal culture, cytology) and can be performed at clinician discretion.

*Response 8. As the CSF picture of TBM varies hugely depending on degree of immunosuppression and ART status we have specified that only one CSF parameter must be abnormal (cell count, protein or glucose). Interpretation of CSF findings, within the clinical picture of the patient, and eligibility for enrolment is at the discretion of the study physician.*

**

*In light of the fact that only around 50% of TBM is microbiologically confirmed and in advanced HIV disease it may be necessary to cover a number of CNS infections empirically whilst waiting for the results of diagnostics tests and imaging, we have gone on to specify in the full protocol that:*

- *Empiric toxoplasmosis therapy is acceptable*
- *Positive CSF or blood cryptococcal antigen relating to prior treated cryptococcal meningitis and receiving secondary prophylaxis is acceptable if microbiologically-confirmed TBM*
- *Study sponsored withdrawal from the trial can take place if there is a microbiological confirmation of another CNS infection other than TBM prior to day 15 of the study*

# Peer review: Reviewer response for version 1

**DOI:** 10.21956/wellcomeopenres.17046.r38567

**Published:** 2020-05-28

**Authors:** Olivier Marcy

**Affiliations:** 1 Centre INSERM U1219, Bordeaux Population Health, University of Bordeaux, Bordeaux, France

This manuscript by Marais, Creswell *et al. *presents an important randomized clinical trial protocol aiming to assess the impact of using high dose oral rifampicin (35mg/kg) versus the usual daily dose of 10 mg/kg in the treatment of tuberculosis (TB) meningitis (TBM). As it is well presented by the authors in the introduction section of the manuscript, evidence is cumulating on the interest of higher doses of rifampicin for pulmonary TB, and the hypothesis is that TBM, the deadliest form of tuberculosis, could benefit from higher oral doses of rifampicin. PK and clinical data from phase 2 studies also confirm the interest of higher doses of rifampicin to decrease the risk of death. As stated by the authors “optimal dose of rifampicin in treatment of TBM should be further investigated in phase III trials”.

 The trial protocol, already approved by national ethics review committees, is globally well presented and as mentioned above very well justified. I have a few comments that should be taken into account to help the reader understand the study.

1. I would suggest mentioning clearly in the rationale or hypothesis why the 35 mg/kg dose was chosen and refer to publications by Boree and collaborators showing the good safety of such high doses.
2. Secondary objectives #6 refers to drug-induced liver injury (DILI) management. When one could expect that occurrence of DILI would differ between the two trial arms despite previous reassuring previous safety data, it is unclear how management itself is an endpoint. One would expect that management of DILI, when it occurs, would be the same in both arms i.e. interruption of potentially hepatotoxic drugs above a determined ALT elevation threshold. Can the authors clarify what they mean by the possible differences in management as an endpoint itself?
3. In their analysis of the primary endpoints, authors will consider loss to follow-up before 8 weeks as a failure (death) and loss to follow-up after 8 weeks as censored data. Although mortality is maximum between the first few weeks of TBM treatment, the probability of survival still decreases steeply beyond 8 weeks. It is therefore very questionable to consider loss to follow-up at any time before 6 months as censored data. I would suggest keeping a more stringent approach considering loss to follow-up as failure at any time and using censoring beyond 8 weeks rather as a sensitivity analysis.
4. Authors should clarify what they mean by adverse events graded 3 to 5 using the Division of AIDS (DAIDS) Table for Grading the Severity of Adult and Pediatric Adverse Events. To my knowledge, the latest version – corrected 2.1 July 2017, and preceding ones only include adverse events graded as severe (3) or life-threatening (4) but no grade 5 events. What adverse events are considered as grade 5 by the investigators? Is it an event leading to death? If it is the case, how will adjudication be done?
5. How extensively will additional active and confirmed CNS infection be investigated? Authors mention CrAg, and microscopy with India ink and gram stain but there seems to be flexibility left to local practices.They should clarify whether local practices include bacteriological culture and investigation for any other viral or fungal infection.
6. The sample size is based on a 50% mortality hypothesis that seems reasonable in an HIV-infected population only but that may appear to be lower in HIV-uninfected patients. Authors should clarify what is the expected HIV prevalence in the study population.
7. The statistical analysis does not include any plan for interim analysis. I recommend clarifying whether the DSMB will be reviewing unblinded data by study arm on the primary endpoint and providing a short description of the interim analyses and stopping rules if any.
8. Of note, another randomized trial will address the question of the effect on TBM mortality of intensified treatment using high dose rifampicin (35 mg/kg) and linezolid with study sites in Uganda and South Africa, as well as Côte d’Ivoire and Madagascar (ClinicalTrials.gov Identifier: NCT04145258). The trial, designed as a factorial trial will also test the addition of low-dose aspirin. I would suggest mentioning this trial in the discussion section and commenting on the different options taken by the 2 trials.

At last, there seems to be a typo when mentioning stratified randomization. I suppose authors are referring to BRC TBM disease grade I or II/III at time of randomization rather than “I or II/II”.

Is the study design appropriate for the research question?

Yes

Is the rationale for, and objectives of, the study clearly described?

Yes

Are sufficient details of the methods provided to allow replication by others?

Yes

Are the datasets clearly presented in a useable and accessible format?

Not applicable

Reviewer Expertise:

TB-HIV co-infection in children and adults, TB meningitis, TB diagnosis in children.

I confirm that I have read this submission and believe that I have an appropriate level of expertise to confirm that it is of an acceptable scientific standard, however I have significant reservations, as outlined above.

# Response

**Published:** 2020-08-03

**Authors:** Fiona Cresswell

**Affiliations:** London School of Hygiene & Tropical Medicine, UK

*We thank the reviewer for their time and expertise in reviewing our protocol manuscript. We have responded to each comment in turn.*

Comment 1. I would suggest mentioning clearly in the rationale or hypothesis why the 35 mg/kg dose was chosen and refer to publications by Boree and collaborators showing the good safety of such high doses.

*We refer to and cite Martin Boeree’s 2015 and 2017 paper in the introduction but have gone onto add further detail about the PanACEA studies in pulmonary TB and the finding of the meta-analysis of Indonesian TBM data.*

*The PanACEA multi-arm multistage trial showed that time to stable culture conversion in liquid media was faster in the 35 mg/kg rifampicin group than in the control group (median 48 days vs 62 days, adjusted hazard ratio 1·78; 95% confidence interval (CI) 1·22–2·58, p=0·003), but not in other experimental arms. In an additional analysis, increasing rifampicin exposure was also associated with shortened time to stable culture conversion. The effect did not plateau, indicating that doses >35 mg/kg could be yet more effective.*

*High-doses rifampicin was also associated with a greater estimated fall in M. tuberculosis bacillary load in sputum of patients with pulmonary TB at 2 weeks follow up, i.e. high-dose rifampicin showed increased ‘early bactericidal activity’. Recent analyses of one of these latter trials (the PanACEA HIGHRIF1 trial) included doses up to 40 mg/kg, demonstrating a further effect of rifampicin exposure on early bactericidal activity and clinical trial simulations showed greater early bactericidal activity for 50 mg/kg rifampicin. Recent evaluations of administration of this 50 mg/kg rifampicin dose show that this dose is not well-tolerated. The maximum tolerable dose for rifampicin in PTB is therefore set at 40 mg/kg daily (R. Aarnoutse and L. te Brake, personal communication, data from the PanACEA HIGHRIF1 trial).* *In summary, data from pulmonary TB have shown that higher rifampicin doses, starting with at least 20 mg/kg daily but certainly at 35 mg/kg orally, result in strongly increased systemic exposures to rifampicin, were safe, tolerated, and resulted in increased response, as reflected in improved early bactericidal activity and shorter time to culture conversion. These lessons learnt in pulmonary TB are relevant to TBM treatment, considering the key role of rifampicin in TBM treatment and the limited CSF penetration seen at standard dose.*

*Further, model-based meta-analysis of data from TB meningitis patients in Indonesia predicted an increase in 6-month survival from approximately 50% to approximately 70% upon increasing the oral rifampicin dose from 10 to 30 mg/kg, and predicted that even higher doses would further improve survival.*

*We have added detail to this effect in the manuscript.*

Comment 2. Secondary objectives #6 refers to drug-induced liver injury (DILI) management. When one could expect that occurrence of DILI would differ between the two trial arms despite previous reassuring previous safety data, it is unclear how management itself is an endpoint. One would expect that management of DILI, when it occurs, would be the same in both arms i.e. interruption of potentially hepatotoxic drugs above a determined ALT elevation threshold. Can the authors clarify what they mean by the possible differences in management as an endpoint itself?

*Thank you for raising this point regarding DILI.* *The aim of the sub-study is to determine the safety of a pragmatic management strategy, and* *does not involve comparison between trial arms.* *Based on safety data from prior Indonesian high dose rifampicin studies it appears that the vast majority (8 of 9) of TBM patients who develop transaminitis (ALT of <10x upper limit of normal, ULN), liver enzymes settle without interruption of anti-TB drugs. Where transaminitis occurs, we therefore plan to closely monitor liver function tests whilst continuing TB treatment unless ALT exceeds a threshold of 10x ULN, at which point we will follow an algorithm of stopping and reintroducing TB drugs sequentially. We have detailed the algorithm for the management of DILI in an SOP. Under trial endpoint #6 we use “management of DILI” as a summary term under which we aim to describe incidence of DILI, frequency and duration of drug interruptions, hepatic outcomes, neurological outcomes.* *We recognise that “management of DILI” is a little non-specific so we have added “incidence and outcome” and will notify IRBs at the time of next regulatory amendments.   * 

Comment 3. In their analysis of the primary endpoints, authors will consider loss to follow-up before 8 weeks as a failure (death) and loss to follow-up after 8 weeks as censored data. Although mortality is maximum between the first few weeks of TBM treatment, the probability of survival still decreases steeply beyond 8 weeks. It is therefore very questionable to consider loss to follow-up at any time before 6 months as censored data. I would suggest keeping a more stringent approach considering loss to follow-up as failure at any time and using censoring beyond 8 weeks rather as a sensitivity analysis.

*This is a point we discussed extensively in the protocol writing process and drew on the experiences at the sites involved in the trial and available literature. We have recently performed a systematic review and meta-analysis of global TBM studies (observational and interventional) which found that over 90% of mortality occurs in the first 12 weeks, with a pooled 12 week mortality of 23% and 12 month mortality of 25% (In press, A Stadelman et al. Open Forum Infectious Diseases). From prior experience at the trial sites loss to follow-up is a rare occurrence (2% in Ugandan meningitis studies) as significant effort is put into tracking patients, supporting outpatient follow up or providing home visits for those who are severely disabled. The rare occurrences of LTFU are usually in confused meningoencephalitis patients who leave hospital against medical advice and without further medical intervention planned, in whom the likelihood of survival is extremely low. We therefore think it is acceptable to keep the censoring point in the protocol at 8 weeks. We will however do an additional sensitivity analysis counting LTFU at any time as an event to see if there is a difference. * 

Comment 4. Authors should clarify what they mean by adverse events graded 3 to 5 using the Division of AIDS (DAIDS) Table for Grading the Severity of Adult and Pediatric Adverse Events. To my knowledge, the latest version – corrected 2.1 July 2017, and preceding ones only include adverse events graded as severe (3) or life-threatening (4) but no grade 5 events. What adverse events are considered as grade 5 by the investigators? Is it an event leading to death? If it is the case, how will adjudication be done?

*Thank you for pointing out this error. Indeed, grade 5 = death. This has been corrected in the manuscript.*

*Comment 5. *How extensively will additional active and confirmed CNS infection be investigated? Authors mention CrAg, and microscopy with India ink and gram stain but there seems to be flexibility left to local practices. They should clarify whether local practices include bacteriological culture and investigation for any other viral or fungal infection.

*The standard of care for routine CSF investigations varies by country and additional tests can be performed beyond the routine depending on clinical circumstances and clinician discretion. All sites include Gram’s stain and bacteriological culture as routine. In all sites, other tests including viral PCR, syphilis serology, fungal culture and cytology can be performed at clinician discretion.*

Comment 6. The sample size is based on a 50% mortality hypothesis that seems reasonable in an HIV-infected population only but that may appear to be lower in HIV-uninfected patients. Authors should clarify what is the expected HIV prevalence in the study population.

*The expected prevalence of HIV in our study population is 60%.*

Comment 7. The statistical analysis does not include any plan for interim analysis. I recommend clarifying whether the DSMB will be reviewing unblinded data by study arm on the primary endpoint and providing a short description of the interim analyses and stopping rules if any.

*Thank you for this suggestion. Detail has been added in the safety section on the DSMB functions, timing, and early termination considerations.*

Comment 8. Of note, another randomized trial will address the question of the effect on TBM mortality of intensified treatment using high dose rifampicin (35 mg/kg) and linezolid with study sites in Uganda and South Africa, as well as Côte d’Ivoire and Madagascar (ClinicalTrials.gov Identifier: NCT04145258). The trial, designed as a factorial trial will also test the addition of low-dose aspirin. I would suggest mentioning this trial in the discussion section and commenting on the different options taken by the 2 trials.

*Thanks for suggesting this. We have added details to the discussion*

Comment 9. At last, there seems to be a typo when mentioning stratified randomization. I suppose authors are referring to BRC TBM disease grade I or II/III at time of randomization rather than “I or II/II”.

*Thank you for spotting this typo! It has been rectified.*
